# Thermal manipulation of the chicken embryo triggers differential gene expression in response to a later heat challenge

**DOI:** 10.1186/s12864-016-2661-y

**Published:** 2016-05-04

**Authors:** Thomas Loyau, Christelle Hennequet-Antier, Vincent Coustham, Cécile Berri, Marie Leduc, Sabine Crochet, Mélanie Sannier, Michel Jacques Duclos, Sandrine Mignon-Grasteau, Sophie Tesseraud, Aurélien Brionne, Sonia Métayer-Coustard, Marco Moroldo, Jérôme Lecardonnel, Patrice Martin, Sandrine Lagarrigue, Shlomo Yahav, Anne Collin

**Affiliations:** URA, INRA, 37380 Nouzilly, France; CRB GADIE, INRA, Domaine de Vilvert, 78350 Jouy-en-Josas, France; GABI, INRA, Plateforme de Microgénomique Iso Cell Express (ICE), 78350 Jouy-en-Josas, France; PEGASE, INRA, Agrocampus Rennes, 35590 Saint-Gilles, France; Institute of Animal Science, The Volcani Center, Bet Dagan, P.O. Box 6, 50250, Israel

## Abstract

**Background:**

Meat type chickens have limited capacities to cope with high environmental temperatures, this sometimes leading to mortality on farms and subsequent economic losses. A strategy to alleviate this problem is to enhance adaptive capacities to face heat exposure using thermal manipulation (TM) during embryogenesis. This strategy was shown to improve thermotolerance during their life span. The aim of this study was to determine the effects of TM (39.5 °C, 12 h/24 vs 37.8 °C from d7 to d16 of embryogenesis) and of a subsequent heat challenge (32 °C for 5 h) applied on d34 on gene expression in the *Pectoralis major* muscle (PM). A chicken gene expression microarray (8 × 60 K) was used to compare muscle gene expression profiles of Control (C characterized by relatively high body temperatures, Tb) and TM chickens (characterized by a relatively low Tb) reared at 21 °C and at 32 °C (CHC and TMHC, respectively) in a dye-swap design with four comparisons and 8 broilers per treatment. Real-time quantitative PCR (RT-qPCR) was subsequently performed to validate differential expression in each comparison. Gene ontology, clustering and network building strategies were then used to identify pathways affected by TM and heat challenge.

**Results:**

Among the genes differentially expressed (DE) in the PM (1.5 % of total probes), 28 were found to be differentially expressed between C and TM, 128 between CHC and C, and 759 between TMHC and TM. No DE gene was found between TMHC and CHC broilers. The majority of DE genes analyzed by RT-qPCR were validated. In the TM/C comparison, DE genes were involved in energy metabolism and mitochondrial function, cell proliferation, vascularization and muscle growth; when comparing heat-exposed chickens to their own controls, TM broilers developed more specific pathways than C, especially involving genes related to metabolism, stress response, vascularization, anti-apoptotic and epigenetic processes.

**Conclusions:**

This study improved the understanding of the long-term effects of TM on PM muscle. TM broilers displaying low Tb may have lower metabolic intensity in the muscle, resulting in decreased metabolic heat production, whereas modifications in vascularization may enhance heat loss. These specific changes could in part explain the better adaptation of TM broilers to heat.

**Electronic supplementary material:**

The online version of this article (doi:10.1186/s12864-016-2661-y) contains supplementary material, which is available to authorized users.

## Background

Broiler chickens are heat sensitive animals. However, their cardiovascular and respiratory organs, and hence their capacity to lose heat via thermoregulatory pathways, have not increased in the same proportions as muscle mass through the 60- year selection process [1]. As poultry production is now widely developed in countries with hot climates such as South America and South East Asia, it is of interest to find new rearing strategies to enhance their thermotolerance [2]. One strategy is to increase the incubation temperature of embryos cyclically in order to induce long-term changes in thermotolerance [3], and several timings and durations of temperature increases have been tested for this purpose [4–8]. A recent study has shown that increasing the incubation temperature from 37.8 to 39.5 °C and relative humidity from 56 to 65 % from days 7 to 16 of embryogenesis for 12 h/d changed body temperature, physiology and parameters of thyroid and adrenal axes, as well as respiratory parameters and stress levels of birds after hatching [9, 10]. This strategy had positive consequences on surviving a heat challenge at slaughter age, i.e. when birds are the most sensitive to heat [9]. However, the molecular determinants of acquisition of such thermotolerance are poorly understood [8]. Some regulatory pathways controlling the plasticity of the central nervous system [11, 12] and epigenetic mechanisms affecting the expression of genes such as the Brain Derived Neurotrophic Factor (BDNF) have been described in postnatal heat-conditioned broilers [13, 14]. It has also been suggested that thermal manipulation during embryogenesis could affect the metabolic rate of chickens [15, 16], and hence their heat production capacity [10, 17, 18]. As the total muscle mass is an essential contributor to the overall heat production in meat-type chickens, we investigated the molecular mechanisms in the tissue that may potentially contribute to the greater adaptability of TM chickens manipulated as embryos when submitted to heat challenge at 34 d.

## Results

In this study, 1000 eggs were incubated in control conditions (C) or in thermally-manipulated conditions (TM). Control eggs were maintained at 37.8 °C and 56 % relative humidity (RH) during the whole incubation period. Thermal manipulation was applied at 39.5 °C and 65 % RH for 12 h/24, from day 7 to day 16 of embryogenesis. At hatching, chickens were reared in a classical pen farm until d34. On d34 half the C (CHC) and half the TM (TMHC) were exposed to heat challenge at 32 °C for 5 h. Eight animals from each treatment, characterized by low body temperatures (Tb) in TM chickens and high Tb in C birds, were slaughtered and the breast muscle was excised, snap-frozen and used for the transcriptomic analysis.

### Quality of data acquisition

In this study, the quality of arrays was found to be good since the expression levels of the control spots present on each slide were similar to those expected and the background noise was relatively low. Furthermore, the arrays were not spotted with single probes and 11,409 of them were repeated at least once (18 %). Most repeated probes corresponding to the same differentially expressed gene with the same annotation (Ensembl, GenBank, etc) were consistently regulated between treatments.

### Numbers of DE genes in each comparison

**Fig. 1:**
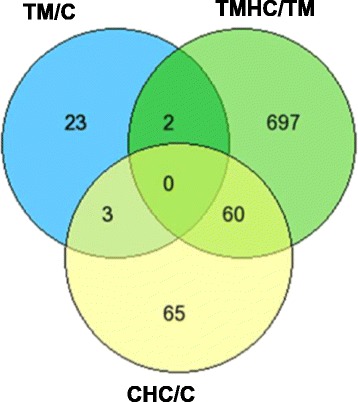
Venn diagram representing the numbers of DE genes in each comparison and DE genes common to several comparisons. No gene was found to be differentially expressed between the three comparisons. Chickens were either incubated and reared in standard conditions (Controls, C), thermally manipulated during embryogenesis and reared in standard conditions (TM), incubated in standard conditions and exposed to heat challenge at d 34 (CHC) or thermally manipulated during embryogenesis and exposed to heat challenge at d 34 (TMHC)

**Fig. 2:**
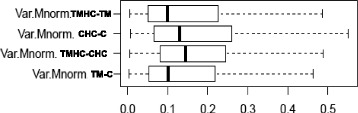
Box plot representing the variance of normalized log-ratios for each comparison. Chickens were incubated and reared in standard conditions (Controls, C), thermally manipulated during embryogenesis and reared in standard conditions (TM), incubated in standard conditions and exposed to heat challenge at d 34 (CHC) or thermally manipulated during embryogenesis and exposed to heat challenge at d 34 (TMHC)

Differentially expressed genes in the four comparisons are represented in a Venn diagram (Fig. 1; http://genevenn.sourceforge.net/). Our results revealed 28 DE genes when comparing TM chickens to controls, but 759 DE genes when comparing TMHC to TM and 128 DE genes when comparing CHC to C. However, no DE gene was found between PM muscles of TMHC and CHC chickens. Few genes (2 to 60) were common between two comparisons, and none was common to the three comparisons. The variance of gene expression was the lowest in the TMHC/TM and TM/C comparisons and the highest in the TMHC/CHC comparison (Fig. 2). However, the numbers of upregulated and down-regulated genes were not different within each comparison (Table 1).

**Table 1:** Numbers of differentially expressed genes (DE) in the *Pectoralis major* muscle in each comparison

Differentially expressed genes	TM/C	CHC/C	TMHC/TM	TMHC/CHC
Upregulated	17	60	417	0
Downregulated	11	68	342	0
Total	28	128	759	0

Chickens incubated and reared in standard conditions (Controls, C), thermally manipulated during embryogenesis and reared in standard conditions (TM), incubated in standard conditions and exposed to heat challenge at d 34 (CHC) or thermally manipulated during embryogenesis and exposed to heat challenge at d 34 (TMHC)

### Real-time PCR validation of a subset of differentially expressed genes arising from the microarray analysis

**Fig. 3:**
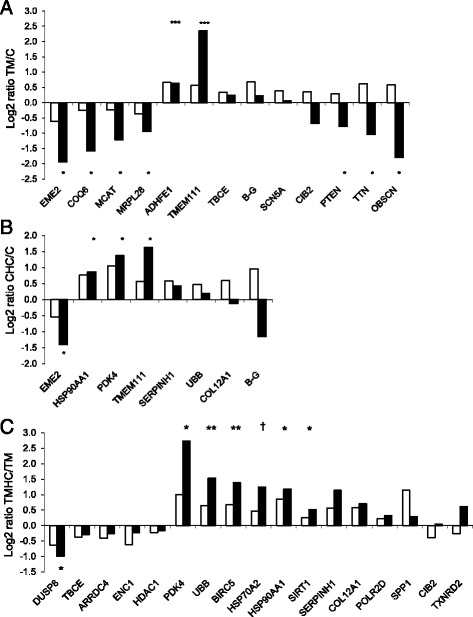
Comparisons of gene expression from microarray and qRT-PCR analysis. Empty bars represent the Log2 ratio obtained from microarray analysis and filled bars represent this value obtained by qRT-PCR. †: *P* < 0.10; *: *P* < 0.05; **P < 0.01; ***P < 0.001. **a** Log2 ratios obtained by comparing chickens thermally manipulated during embryogenesis and reared in standard conditions (TM) to control chickens incubated and reared in standard conditions (**C**). **b** Log2 ratios obtained by comparing control chickens submitted to a heat challenge at day 34 (CHC) to C chickens. **c** Log2 ratios obtained by comparing TM chickens submitted to a heat challenge at day 34 (TMHC) to TM chickens. EME2, essential meiotic endonuclease 1 homolog 2; COQ6: coenzyme Q6 homolog, monooxygenase; MCAT: malonyl CoA:ACP acyltransferase; MRPL28: mitochondrial ribosomal protein L28; ADHFE1: alcohol dehydrogenase, iron containing, 1; TMEM111: transmembrane protein 111; TBCE: tubulin folding cofactor E; B-G: V-region-like B-G antigen-like; SCN5A: sodium channel, voltage-gated, type V, alpha subunit, transcript variant 4; CIB2: calcium and integrin binding family member 2; PTEN: phosphatase and tensin homolog; TTN: titin; OBSCN: obscurin, cytoskeletal calmodulin and titin-interacting RhoGEF; HSP90AA1: heat shock protein 90 kDa alpha (cytosolic), class A member 1; PDK4: pyruvate dehydrogenase kinase, isozyme 4; SERPINH1: serpin peptidase inhibitor, clade H (heat shock protein 47), member 1; UBB: ubiquitin B; COL12A1: collagen, type XII, alpha 1; DUSP8: Dual specificity phosphatase 8; ARRDC4: arrestin domain containing 4; ENC1: ectodermal-neural cortex 1; HDAC1: histone deacetylase 1; BIRC5: baculoviral IAP repeat-containing 5 (survivin); HSP70A2: Heat shock protein 70 A2; SIRT1: sirtuin 1; POLR2D: polymerase (RNA) II (DNA directed) polypeptide D; SPP1: secreted phosphoprotein 1; TXNRD2: thioredoxin reductase 2

The expression of 28 DE genes selected in the TM/C comparison (EME2, COQ6, MCAT, MRPL28, ADHFE1, TMEM111, TBCE, B-G, SCN5A, CIB2, PTEN, TTN, OBSCN), the in CHC/C comparison (EME2, HSP90AA1, PDK4, TMEM111, SERPINH1, UBB, COL12A1, B-G) and the TMHC/TM comparison (DUSP8, ARRDC4, ENC1, HDAC1, PDK4, UBB, BIRC5, HSP70A2, HSP90AA1, SIRT1, SERPINH1, COL12A1, POLR2D, SPP1, CIB2, TXNRD2) was analysed by real-time quantitative PCR (Fig. 3). In the TM/C comparison (Fig. 3a), nine of 13 genes were confirmed as DE, of which six genes (EME2, COQ6, MCAT, MRPL28, ADHFE1, TMEM111) were upregulated or down-regulated with both techniques, whereas the three remaining DE genes (PTEN, TTN, OBSCN) were upregulated in TM with microarray and down-regulated in TM with PCR (Fig. 3a). In the CHC/C comparison (Fig. 3b), the significant difference between treatments was validated for four genes (EME2, HSP90AA1, PDK4, TMEM111), all with consistent patterns of expression between qPCR and array results. Finally, in the TMHC/TM comparison (Fig. 3c), 15 out of 17 genes showed consistent expression difference between treatments with both techniques (microarray and real time RT-PCR) but the difference was significantly (*P* < 0.10) validated by PCR for only seven of them.

### Gene ontology associated with DE genes in each comparison

#### TM/C comparison

When analysing the ontology of the 28 DE genes of the TM/C comparison, 16 genes were classified using Genomatix software (Table 2). Six genes were found to be involved in energy metabolism and mitochondrial functioning (5 down-regulated: MRPL28, COQ6, MCAT, ACP6, CYP39A1 and one upregulated: TBL1X), and three genes were involved in cell proliferation and vascularization (BTC, ABHD2 and EPS15, all upregulated); four genes are known to be involved in muscle growth (MBNL2, RBM20, ADHFE1 being upregulated and SLC25A33 down-regulated). Finally, one gene encodes a receptor for pain (P2RX4, down-regulated) and EME2 (down-regulated) is involved in DNA repair.

**Table 2:** Gene onthology of the TM/C DE genes

Gene name	Molecular function	Fold Change
Energy metabolism		
MRPL28	Mitochondrial ribosomal protein encoded by the nucleus which helps in protein synthesis within the mitochondrion	0.78
COQ6	Monooxygenase required for the biosynthesis of coenzyme Q10.	0.84
MCAT	Enzyme which catalyzes the transfer of a malonyl group from malonyl-CoA to the mitochondrial acyl carrier protein.	0.85
TBL1X	Corepressor SMRT (silencing mediator for retinoid and thyroid receptors) complex along with histone deacetylase 3 protein.	1.28
ACP6	Phosphatase which regulates lipid metabolism in mitochondria.	0.81
CYP39A1	Monooxygenase, which catalyzes many reactions involved in drug metabolism and synthesis of cholesterol, steroids and other lipids.	0.84
Cell proliferation and vascularization pathway		
BTC	Ligand for the EGF receptor, regulation cell growth and differentiation.	1.22
ABHD2	Protein specifically expressed in mouse vascular muscle cells and not in skeletal muscle cells.	1.24
EPS15	Protein involved in EGFR pathway.	1.24
Muscle growth		
MBNL2	C3H-type zinc finger protein that modulates alternative splicing of pre-mRNA, involved in the physiopathology of muscle dystrophy.	1.25
RBM20	RNA binding protein involved in familial dilated cardiomyopathy and is involved in alternative splicing of TTN.	1.37
TTN	Protein of striated muscle.	1.53
OBSCN	Proteins that include titin and nebulin, and may have a role in the organization of myofibrils during assembly and may mediate interactions between the sarcoplasmic reticulum and myofibrils.	1.50
ADHFE1	Hydroxyacid-oxoacid transhydrogenase which is responsible for the oxidation of 4-hydroxybutyrate in mammalian tissues; involved in increased muscle mass in horses.	1.58
SLC25A33	Pyrimidine Nucleotide Carrier which is associated with muscle wasting.	0.82
B-G	Leukocyte antigen.	1.59
Receptor for pain		
P2RX4	Purinoreceptor for ATP-gated ion channel, involved in pain reception in mouse.	0.78
Ion Channel		
SCN5A	Integral membrane protein and voltage-gated sodium channel subunit.	1.31
DNA repair		
EME2	Involved in repairing DNA damage and maintaining genomic stability.	0.65

MRPL28, Mitochondrial ribosomal protein L28; COQ6, Coenzyme Q6 homolog, monooxygenase; MCAT, Malonyl CoA:ACP acyltransferase; TBL1X, Transducin (beta)-like 1X-linked; ACP6, acid phosphatase 6; CYP39A1, Cytochrome P450, family 39, subfamily A, polypeptide 1; BTC, Betacellulin, ABHD2, Abhydrolase domain containing 2; EPS15, Epidermal growth factor receptor pathway substrate 15; MBNL2, Muscleblind-like 2 Drosophila; RBM20, RNA binding motif protein 20; TTN, Titin; OBSCN, Obscurin; ADHFE1, Alcohol dehydrogenase, iron containing, 1; SLC25A33, Carrier family 25, member 3; B-G, V-region-like B-G antigen-like; P2RX4, purinergic receptor P2X, ligand-4; SCN5A, sodium channel, voltage-gated, type V, alpha subunit, transcript variant 4; EME2, Essential Meiotic endonuclease 2 homolog 1

#### CHC/C comparison

One hundred and twenty-eight genes were differentially expressed in the PM muscle between CHC and C birds. Fifty-six, among which half were common to the TMHC/TM comparison, were associated with metabolic regulation (*P* < 0.01), 25 were associated with the stress response (among which half were common to the TMHC/TM comparison, *P* < 0.01), seven are related to RNA metabolism and splicing (*P* < 0.01), six to the steroid hormone response (*P* < 0.01) and six to the regulation of protein metabolism (*P* < 0.01; Table 3). The ten most highly upregulated and down-regulated genes in the CHC/C comparison are reported in Table 4.

**Table 3:** Functions of differentially expressed CHC/C genes in Pectoralis major muscle

Biological pathways	Differentially expressed genes between CHC and C	P value
RNA metabolism and splicing	14, of which 7 common with TMHC/TM	3.80e-5, 1.13e-3
Vascularization and regulation of arterial pressure	7, of which 1 common with TMHC/TM	1.29e-3, 3.01 e-3
Stress response	25, of which 12 common with TMHC/TM	1.61e-3, 5.40e-3
Regulation of protein metabolism	6, of which 2 common with TMHC/TM	1.58e-3, 2.07e-3
Steroid hormone response	6, of which 1 common with TMHC/TM	3.84e-3
Metabolic regulation	56, of which 26 common with TMHC/TM	5.59e-3, 9.38e-3

**Table 4:** Top 10 DE genes downregulated and upregulated in CHC/C comparison

Accession Number	Gene Name	Gene description	Fold Change
Top ten down-regulated DE genes in CHC/C list			
NM_001031347	CIRBP	Cold inducible RNA binding protein	0.48
XM_419942	CPSF3	Similar to Cleavage and Polyadenylation Specifity Factor protein	0.57
NM_001031044	DUSP10	Dual specificity phosphatase 10 (DUSP10), mRNA [NM_001031044]	0.61
XM_414105	SIAH1	E3 Ubiquitin Protein Ligase 1	0.61
XM_419574	C1orf96	Chromosome 1 open reading frame 96	0.64
XM_001232892	DUSP8	Dual specificity phosphatase 8	0.66
NM_001031197	SRSF5	Serine/arginine-rich splicing factor 5	0.66
XM_003642723	DYRK3	Dual-specificity tyrosine-(Y)-phosphorylation regulated kinase 3, transcript variant 1	0.66
XM_417975	DYRK3	Dual-specificity tyrosine-(Y)-phosphorylation regulated kinase 3, transcript variant 2	0.66
XM_003642238	RNF39	Zinc finger protein RFP-like	0.67
Top ten up-regulated DE genes CHC/C list			
XM_001233913	FAM188B2-like	Family 188 B2-like transcript	5.21
NM_001199909	PDK4	Pyruvate dehydrogenase kinase, isozyme 4	2.06
NM_001031810	B-G	MHC B-G antigen	1.95
NM_205021	COL12A1	Collagen, type XII, alpha 1	1.75
XM_418238	COMP	Cartilage oligomeric matrix protein	1.70
NM_001109785	HSP90AA1	Heat shock protein 90 kDa alpha class A member 1	1.69
NM_205456	TNC	Tenascin C	1.60
NM_205291	SERPINH1	Serpin peptidase inhibitor, clade, member 1	1.54
NM_001012576	HSPA4L	Heat shock 70 kDa Protein 4-Like	1.52
NM_204289	HSP90B1	Heat shock Protein 90 kDa beta member 1	1.48

#### *TMHC/TM* comparison

A much higher number of DE genes were found in this comparison, among which the largest groups were involved in metabolic processes (293 genes, *P* < 0.01), macromolecule metabolism (202, *P* < 0.01) and the stress response (98, *P* < 0.01). Other DE genes in this comparison were associated with the organization of organelles (82, *P* < 1.00e^-5^), the cell cycle (59, *P* < 0.01), RNA metabolism and splicing (44, *P* < 0.01), proteolytic metabolism (33, *P* < 0.01), chromatin organization (26, *P* < 0.01), vascularization and the regulation of arterial pressure (25, *P* < 0.01), endopeptidase activity (13, *P* < 0.01), skeletal muscle development (10, *P* < 0.01) and monocarboxylic acid metabolism (8, *P* < 0.001; Table 5). Among these, most of the groups shared fewer than 10 % DE genes in common with the CHC/C comparison. The genes included in the ontology items related to chromatin organization, skeletal muscle development, vascularization and the regulation of arterial pressure (except for one gene) were specific to the comparison between both TM groups. The ten most highly upregulated and down-regulated genes in the TMHC/TM comparison are summarized in Table 6.

**Table 5:** Functions of differentially expressed TMHC/TM genes in Pectoralis major muscle

Biological pathways	Differentially expressed genes between TMHC and TM	*P* value
Cellular cycle/ Mitosis/ Organelles fission/ M phase	59, of which 4 common with CHC/C list	8.63e-3-2.56e-7
RNA metabolism and splicing	44, of which 7 common with CHC/C	6.67e-7, 1.23e-5
Organization of organelles	82, of which 6 common with CHC/C	5.20e-6
Macromolecule metabolism	202, of which 22 common with CHC/C	1.05e-3
Metabolic process	293, of which 25 common with CHC/C	4.25e-4, 2.07e-3
Protolithic mechanisms	33, of which 2 common with CHC/C	1.54e-3-8.73e-3
Vascularization and regulation of arterial pressure	25, of which 1 common with CHC/C	1.61e-3, 6.31e-3
Stress response	98, of which 12 common with CHC/C	1.83e-3
Chromatin organization, remodeling, and silencing	26, of which 0 common with CHC/C	1.91e-3, 8.42e-3
Development of skeletal muscle	10, of which 0 common with CHC/C	8.14e-3, 8.19e-3
Regulation of endopeptidase activity	13, of which 4 common with CHC/C	3.88e-3
Cell death and apoptosis	65, of which 3 common with CHC/C	6.03e-4, 3.35e-3
Monocarboxylic acid metabolism	8, of which 4 common with CHC/C	7.79e-4

**Table 6:** Top 10 DE genes downregulated and upregulated in TMHC/TM comparison

Accession Number	Gene Name	Gene description	Fold Change
Top ten down-regulated DE genes in TMHC/TM list			
XM_001233913	FAM188B2-like	Family 188 B2-like transcript	0.20
NM_001031347	CIRBP	Cold inducible RNA binding protein	0.45
NM_205217	ST3GAL1	ST3 beta-galactoside alpha-2,3-sialyltransferase 1	0.53
XM_419574	C1orf96	Similar to Chromosome 1 open reading frame 96	0.56
XM_424790	ENC1	Ectodermal-neural cortex 1	0.56
NM_001031197	SRSF5	Serine/arginine-rich splicing factor 5	0.56
XM_003642257	SLC25A25	Solute carrier family 25, member 25	0.57
NM_001031197	SFRS5	Splicing factor, arginine/serine-rich 5	0.58
NM_001031499	YOD1	YOD1 OTU deubiquinating enzyme 1 homolog	0.59
Top ten up-regulated DE genes TMHC/TM list			
NM_204535	SPP1	Secreted phosphoprotein 1 Osteopontin	2.37
NM_001199909	PDK4	Pyruvate dehydrogenase kinase, isozyme 4	1.99
NM_001159698	HSPH1	Heat shock 105 kDa/110 kDa protein 1	1.93
NM_001277769	CLDN10	Claudin 10	1.92
NM_001109785	HSP90AA1	Heat shock protein 90 kDa alpha class A member 1	1.81
NM_205125	DKK3	Dickkopf homolog 3	1.76
NM_205003	HSPA8	Heat shock 70 kDa protein 8	1.73
XM_004946671	UBC	Ubiquitin C	1.72
NM_001006278	MMP7	Matrix metallopeptidase 7	1.69
XM_413746	DNAJA4	DnaJ (Hsp40) homolog, subfamily A, member 4	1.67

### Clustering

**Fig. 4:**
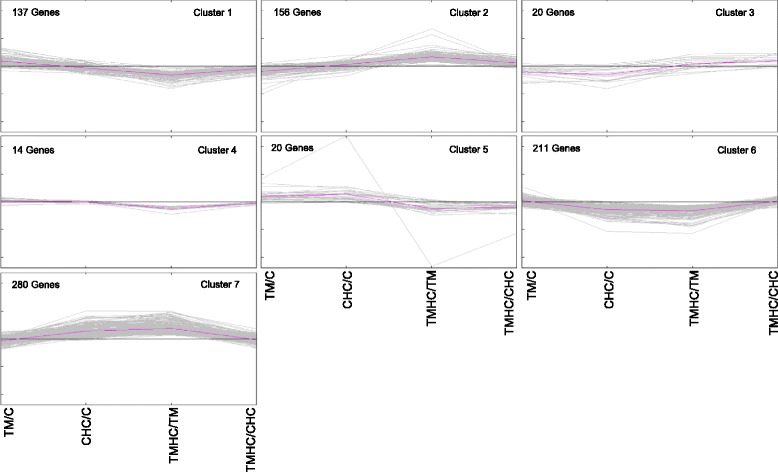
Clusters resulting from the hierarchical clustering of DE genes using Pearson correlation coefficient. Seven clusters were obtained from the expression profiles of DE genes in at least one comparison. Chickens were incubated and reared in standard conditions (Controls, C), thermally manipulated during embryogenesis and reared in standard conditions (TM), incubated in standard conditions and exposed to heat challenge at d 34 (CHC) or thermally manipulated during embryogenesis and exposed to heat challenge at d 34 (TMHC). Clusters were generated with MeV software

Genes that were DE in at least one comparison were clustered to visualize similar expression profiles (Fig. 4). The clustering of DE genes resulted in seven clusters with similar expression profiles between conditions with 137, 156, 20, 14, 32, 211 and 280 genes per group for clusters 1 to 7, respectively. Clusters 2 and 3 presented genes over expressed in C compared to TM and in TMHC compared to TM, respectively. Cluster 5 was especially interesting as DE genes in this cluster exhibited opposite responses of C and TM chickens to heat challenge. In contrast, both C and TM in clusters 6 and 7 showed similar responses to heat challenge, being respectively downregulated and upregulated compared to their respective controls. Gene classification by clustering also enabled to determine the upstream regulator responsible for the expression profile. Upstream regulators per cluster are presented in Additional file 1.

## Discussion

The aim of this study was to determine the long-lasting effects of, TM during chicken embryogenesis associated with lower body temperatures, and of a subsequent heat challenge, on muscle gene expression in 34-day-old broilers. In standard rearing conditions (21 °C), TM significantly modified the expression of only 28 genes, demonstrating a relatively low impact of this embryonic treatment on muscle gene expression under control conditions. However, the heat challenge changed gene expression profiles in TM broilers to a much greater extent than in control birds, with 759 and 128 genes differentially expressed between TMHC and CHC, respectively. This result might reflect better adaptability of TM chickens characterized by low Tb to respond to acute heat challenge. In our conditions, we did not find any DE genes in the TMHC/CHC comparison, probably partly due to the higher variance of expression reported in this comparison than that in the others. Indeed, exposure to high temperature may have triggered differences between animal responses, inducing more variability in production traits, but also in gene expressions. Such hypothesis was suggested by Debut et al. [19] considering variations of chicken technological meat quality in relation to preslaughter stress conditions. Consistently, previous results indicated around 20 % higher coefficients of variation in the expression of candidate genes in the muscle of birds in the fed state submitted or not to heat at 5 weeks of age [18, 20].

A recent study analysing the effects of chronic heat on global muscle gene expression in 28-day-old chickens [21] revealed only two DE genes that were also DE in the present analysis: Ubiquitin C (UBC) and pyruvate dehydrogenase kinase-like protein (PDK4) that were upregulated during the heat challenge (common to CHC/C and TMHC/TM lists of DE genes). UBC is involved in proteolysis via the ubiquitin-proteasome pathway, a biological process activated by high ambient temperature, whether directly or as a consequence of reduced feed intake [20]. PDK4 is a protein located in the matrix of mitochondria that inhibits the pyruvate dehydrogenase complex by phosphorylating one of its subunits, thereby contributing to the regulation of glucose metabolism [22]. Interestingly, glucose metabolism was previously demonstrated to be affected by heat exposure in broiler chickens, suggesting a lower capacity of insulin to stimulate glucose uptake or delayed response kinetics under heat conditions [20]. Among the DE genes responding to heat challenge conditions in both control and thermal manipulated chickens, genes involved in cell signalling and the cell stress response were found in particular. The most down-regulated gene was CIRBP (Cold-Induced RNA Binding Protein) that was common in the CHC/C and TMHC/TM comparisons. This gene is involved in the regulation of the circadian clock and its expression has already been shown to be upregulated in mice exposed to hypothermia conditions [23], and downregulated when germ cells were exposed to elevated temperatures [24]. In our study, Fam188-B2-like was another gene that was highly regulated in response to heat. Its expression was increased in controls and decreased in TM in response to heat. This gene may be an interesting marker for thermotolerance but its function is not yet known and requires further analysis.

### Changes in energy metabolism, muscle growth and development

**Fig. 5:**
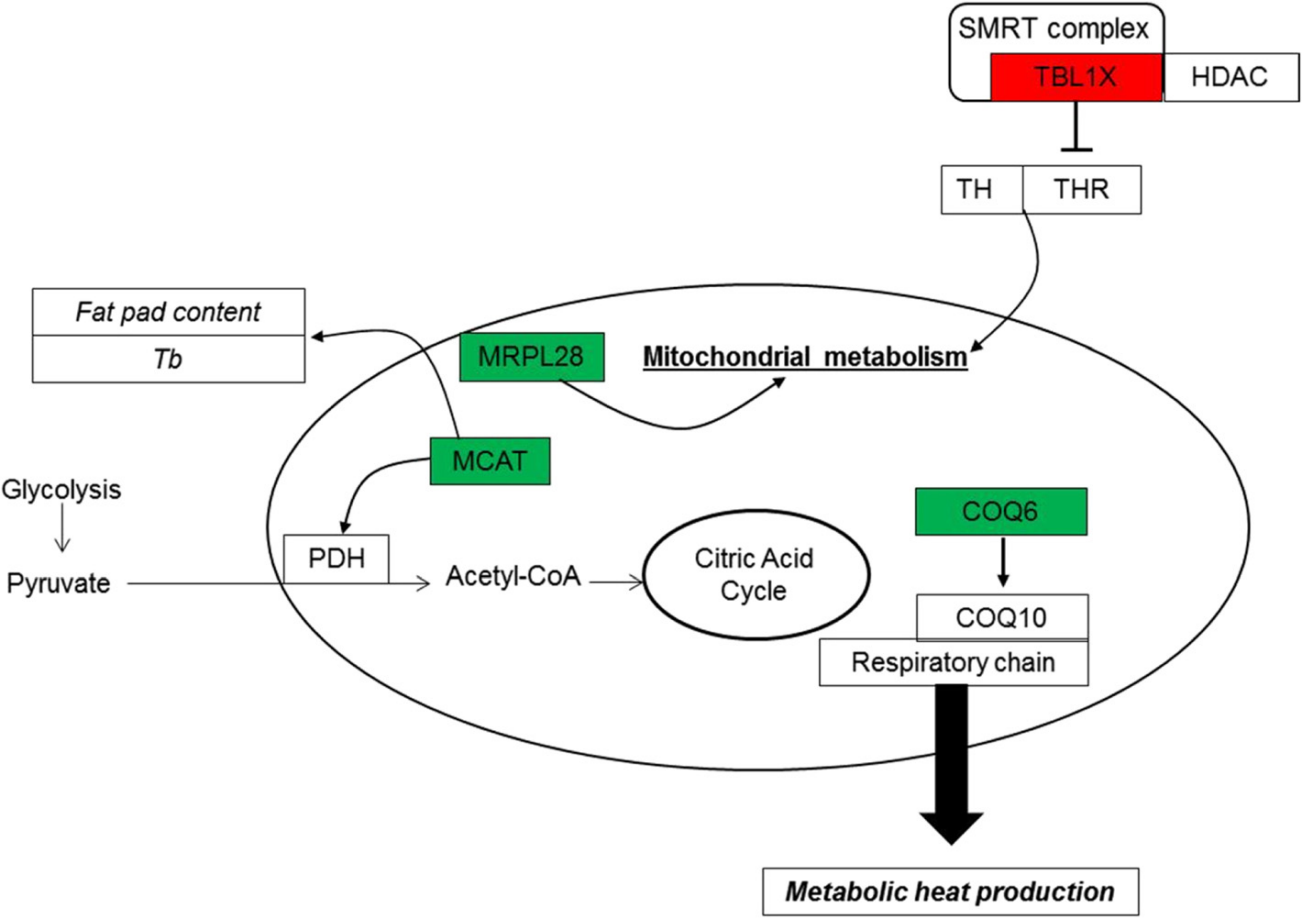
Summary of interactions between genes differentially expressed between TM and C muscles in relation to mitochondrial functioning. Genes highlighted in red and green were up- and down-regulated in TM compared to C muscles, respectively. Gene names are indicated in capitals according to Gene Ontology. Chickens were incubated and reared in standard conditions (Controls, C) or thermally manipulated during embryogenesis and reared in standard conditions (TM). COQ6: coenzyme Q6 homolog, monooxygenase; COQ10: coenzyme Q10 homolog; HDAC: histone deacetylase; TBL1X: transducin (beta)-like 1X-linked; SMRT: silencing mediator for retinoid and thyroid receptors; TH: thyroid hormone; THR: thyroid hormone receptor; PDH: pyruvate dehydrogenase; MCAT: malonyl CoA:ACP acyltransferase; MRPL28: mitochondrial ribosomal protein L28; Tb: body temperature

Piestun et al. [16] showed that O_2_ consumption decreased after TM of chicken embryos, suggesting a lower intensity of energy metabolism and subsequent metabolic heat production in TM birds. Furthermore, thyroid hormones involved in the regulation of these pathways [25, 26] have been reported to be modified by TM in standard rearing conditions [9, 10]. In the TM/C comparison, four DE genes are involved in mitochondrial energy production (Fig. 5). TBL1X, which was upregulated in the muscle of TM chickens, encodes transducin, a subunit of the transcriptional corepressor SMRT (silencing mediator for retinoid and thyroid hormone receptors) complex that also includes histone deacetylase 3 protein [27]. The increased expression of TBL1X may be a long-term response to TM, contributing to reduced mitochondrial energy metabolism and subsequent metabolic heat production. Moreover, local muscle thyroid metabolism investigated by deiodinase expression seemed to be affected in 34-day-old broilers [18]. Consequently, TM may have induced a decrease in both the peripheral thyroid hormone metabolism and the thyroid hormone receptor availability, possibly resulting in lower energy metabolism and heat production. Moreover, we observed in a previous study that TM during embryogenesis induced a long-term decrease in PGC-1α (a transcriptional cofactor enhancing mitochondrial biogenesis and activity) under standard conditions [18]. In addition, MCAT expression was reduced in TM compared to C, possibly decreasing the oxidation of pyruvate. MCAT has been shown to be involved in the activation of pyruvate dehydrogenase, an enzyme involved in the mitochondrial oxidative decarboxylation of pyruvate into acetyl-coA, which is then integrated in the citric acid cycle. Interestingly, MCAT-deficient mice were found leaner that controls, exhibited hypothermia and diminished activity of the citric acid cycle and energy metabolism [28]. Furthermore, COQ6 expression in the muscle was decreased in TM compared to C chickens. COQ6 is required for the biosynthesis of COQ10 which is an essential component of the mitochondrial respiratory chain. Taken together, these results suggest that TM chickens characterized by low Tb may have lower respiratory chain activity and thus decreased heat production. Another marker of mitochondrial activity involved in mitochondrial proteosynthesis, MRPL28, was significantly decreased by TM. MRPL28 knockdown has been reported to induce a decrease in mitochondrial activity in pancreatic tumour cells [29]. These findings corroborate the hypothesis of decreased metabolic activity in TM birds in standard rearing conditions, thus reducing metabolic heat production.

In addition to these modifications of gene expression in muscle associated with TM treatment, changes in gene response resulting from heat challenge during the whole life span were observed. Heat exposure of broilers is reported to result in several physiological and behavioural changes intended to restore homeostasis by reducing their resting metabolic rate [30]. To cope with high environmental temperatures, broilers tend to increase thermolysis and to down-regulate heat-producing pathways. Muscles are able to exhibit differential gene expression profiles in response to heat exposure, due to their involvement in metabolic heat production explained by their relative mass to body weight (around 50 % of the carcass weight for breast and leg muscles [31]). In the gene ontology study, the two most important categories in the TMHC/TM comparison were “macromolecule metabolism” and the “metabolic process”, with 202 and 293 DE genes, respectively. For instance, one of the most strongly down-regulated genes in this comparison was SLC25A25, an ATP/Mg2+ Pi transporter involved in energy efficiency that is highly overexpressed in the skeletal muscle of UCP1-deficient mice under cold conditions [32].

Furthermore, relatively high numbers of mRNA samples encoding mitochondrial tRNA synthases or ribosomal proteins were found to be down-regulated in the TMHC/TM comparison (data not shown), which could contribute to limitations in synthesis of mitochondrial protein (especially subunits of the respiratory chain) specifically in TM birds characterized by low Tb in response to heat exposure. Thermal manipulation during embryogenesis thus may have an effect on the expression (activation or inhibition) of a significant number of metabolic genes in PM muscle, possibly regulating metabolic heat production and thus allowing birds to better adapt to high environmental temperatures.

Parallel with these metabolic adaptations, the mechanisms regulating muscle growth and development seemed to be affected in TM broilers displaying low Tb. Previous studies also suggested that TM increases breast muscle yield [10, 16] and regulates pathways involved in muscle growth and development [18]. In the TM/C comparison, we observed the up-regulation of genes potentially associated with increased muscle mass (*ADHFE1, MBNL2, TMEM111*; [33–35]) or the down-regulation of a gene (*SLC25A3*) associated with muscle wasting [36]. TM during embryogenesis performed under our conditions may therefore have had a lasting effect on energy metabolism and heat production, thus modifying muscle growth and development. These modifications may limit heat-producing mechanisms when chickens are exposed to high temperatures at slaughter age, while preserving muscle growth.

### Modifications of vascularization

Recent studies have hypothesized that TM may affect vascularization in the chicken in the long term, or may adapt chicken vascularization to facilitate heat loss during heat challenge [8, 37]. Pinchuk et al. [37] observed that TM enhanced the vascular density of the chorio-allantoic membrane (CAM) during development, and increased the expression of angiogenic genes (*VEGF, FGF, HIF-1α and MMP-2*). Interestingly, our study also showed that TM chickens characterized by low Tb developed pathways involved in vascularization and angiogenesis.

At 21 °C, we found three pro-angiogenic upregulated genes in the TM compared to C chickens (*ABHD2, EPS15 and BTC*). Even in standard rearing conditions, an increase in the expression of pro-angiogenic genes might prepare chickens for further environmental temperature changes by modifying vascularization properties to facilitate heat dissipation as an adaptive mechanism.

In agreement with this, this pathway also seemed to be specifically affected by heat challenge in TM animals displaying low Tb. Indeed, during acute heat stress, 25 DE genes in the TMHC/TM comparison are involved in angiogenesis and vascularization, with only one gene in common with the CHC/C comparison. This result suggests that angiogenesis and/or vascularization were modified when TM chickens were exposed to heat challenge, further favouring heat dissipation. Blood circulation from skeletal muscle to the skin may be critical to ensuring sensible heat loss. Within the upregulated genes, 11 are directly involved in angiogenesis and vascularization (*AGTR1, THY1, AGTR2, TGFBR1, HMOX1, BTG1, ANGPT2, MMP2, SERPINF2, AVPR1B, and FGF18*). Another group of eight upregulated DE genes is involved in reorganisation of the extracellular matrix (ECM; *ITGAV, GJA1, PCSK5, COL4A3, COL1A2, COL5A1, CDH2, FN1*). The ECM has an important role in angiogenesis by providing the structural support necessary for blood vessel formation and supplying several endogenous pro- and anti-angiogenic factors that regulate endothelial cell survival and vessel stability [38]. A higher level of expression of genes involved in ECM reorganisation may thus facilitate expansion of vascularization in TM chickens.

When comparing the upstream regulators of TMHC/TM and CHC/C DE gene lists using ingenuity pathway analysis (IPA), the protein regulator vascular endothelial growth factor (VEGF) was found to be a predicted activated upstream regulator of 25 genes specific to the TMHC/TM gene list (but not the CHC/C gene comparison). VEGF is a cellular signal protein that stimulates vasculogenesis and angiogenesis by activating proliferation and migration of endothelial cells [39]. The expression of this gene is increased in the chorio-allantoic membrane of TM embryos [37]. The data also supported a strong involvement of the transforming growth factor β1 (TGFβ1) pathway, as this gene was an upstream regulator of 61 genes in the TMHC/TM list and of 12 genes of the CHC/C list (Additional file 1). TGFβ1 is involved in the formation of new functional microvascular networks [40].

Altogether, these results indicate that TM associated with low Tb induced changes in the regulation of broiler vascularization and angiogenesis, observed under standard rearing conditions (TM/C comparison), and following heat stress (TMHC/TM comparison) when it could favour a vasomotor response to heat.

### Stress response and apoptosis

This study also revealed changes in pathways regulating stress responses and apoptosis in TM chickens characterized by low Tb. Indeed, P2RX4, a purinergic receptor, was downregulated in TM chickens compared to C chickens. A reduction in chronic and acute pain was observed in P2RX4-/- mice [41], and such downregulation may have resulted in lower sensitivity of TM broilers to heat, thus minimizing the effects of high ambient temperature, especially in the PM muscle.

When performing the gene ontology analysis, the stress response was a significant pathway in both comparisons of heat challenged chickens and their controls (CHC vs C and TMHC vs TM). The expression of 13 heat shock proteins (HSP) and chaperone proteins was modified in the TMHC/TM comparison, whereas expression of only three HSP was modified in CHC/C. These proteins act as chaperone proteins protecting cell integrity from heat stress and protein degradation. The expression of HSP90AB1 was considerably lower in TMHC than in TM birds, as confirmed by real time RT-PCR. A polymorphism in this gene has been associated with heat tolerance in indigenous Thai cattle [42]. In the TMHC/TM DE gene list related to the stress response category, 36 genes are anti-apoptotic while 15 are pro-apoptotic. In the CHC/C list, six have been shown to have an anti-apoptotic effect and nine a pro-apoptotic effect. These results suggest that TM chickens characterized by lower body temperature than C chickens may have lower heat-sensitivity and be better adapted to heat, and have developed chaperone protection and enhanced anti-apoptotic pathways under heat stress. In addition to having pathways regulating metabolism, vascularization and cell fate, epigenetic pathways in TM animals displaying low Tb were particularly affected by heat challenge.

### Epigenetic mechanisms as a molecular basis for differences in gene expression

The TMHC/TM comparison revealed 759 DE genes compared to 128 in the CHC/C comparison. One hypothesis to explain this difference is that TM animals that had already experienced heat during the incubation period, could have setup more diverse or more reactive adaptive mechanisms than control birds during their development, improving their adaptive capacity while later submitted to heat challenge. The first experience of heat, during embryogenesis, may have modified the threshold response to heat or have induced better plasticity to heat response in later life [11]. In this study, we observed differential expression of 26 genes involved in chromatin organization, remodelling and gene silencing specific to the TMHC/TM comparison. Most of them are involved in modifications of chromatin conformation (*SIRT1, RUVBL1, CHAF1B, INO80, MYST4, ATXN7, KAT2A, MBIP, CHD1, BANP, CDYL, ARID4B, NURD, SIN3A* Complex*, UBE2B, WHSC1, DOT1L, SETD2, HMTs*). These epigenetic modifiers were specifically affected by heat challenge in TM animals, but not in controls. This possible remodelling induced by heat exposure during embryogenesis may have modified the dynamics of chromatin architecture to allow access of the regulatory transcription machinery, thereby controlling gene expression to favour efficient response of TM birds to heat. The details of these mechanisms remain to be elucidated. However, chromatin modification has already been shown to be responsible for metabolic plasticity in response to environmental changes, thus permitting rapid adaptation of physiological processes [43]. Thermal manipulation and possibly the later heat challenge may have modified the chromatin landscape compared to the controls, since metabolic and environmental stimuli have been shown to have critical roles in determining chromatin structure [44]. Thermal manipulation applied 3 d post-hatch in chicks has been reported to induce modification of the expression of BDNF (brain-derived neurotrophic factor), which encodes a key regulator of thermotolerance in the chick hypothalamus [13]. This was associated with epigenetic modifications such as changes in the methylation level of CpG sites in the promoter of the BDNF gene. Modification of histone H3 lysine 9 (H3K9) and methylation of histone H3 lysine 27 (H3K27) in the promoter of BDNF occurred in these conditions*,* probably changing the threshold response to heat [45].

In the present experiment, 44 DE genes in the TMHC/TM comparison are involved in mRNA metabolic processing, transcription degradation and splicing, which could further control gene expression in TM birds characterized by low Tb when exposed to heat.

### Cluster analysis and potential upstream regulators

The cluster study revealed potential upstream regulators for the group profile (Fig. 4). Interestingly, for cluster 2 (genes with increased expression during heat stress in TM broilers), Forkhead box protein M1 (FOXM-1) was a candidate upstream regulator displaying a highly significant *P*-value of overlap (*P* = 4.51e^-11^). FOXM-1 has already been involved in thermal resistance in humans [46]. Cluster 5, exhibiting opposite responses of C and TM chickens to heat challenge, comprised only 20 genes. The upstream analysis of this cluster revealed immune system cytokines as regulators of some of the genes: TNF for 5 genes, IL1- β for 3 genes and IL3 for 2 genes. A recent study demonstrated that heat-stress inhibited TNF-α and IL1-β expression and increased interleukins IL-6 and IL-10 expression in the muscle, as an innate immune response to heat [47]. Immune or inflammatory responses to heat stress may have been modified in TM broilers that had already experienced heat exposure during embryogenesis, probably changing the expression of downstream genes.

## Conclusions

In this study we identified long-term modifications of physiological regulators induced by TM during embryogenesis and a subsequent effect of heat challenge in the PM muscle of 34-day-old broilers. The results of this study indicated new pathways involved in heat adaptation of TM chickens characterized by low body temperature. First of all, our results in broiler chickens reared in standard conditions corroborate the hypothesis of reduced energy metabolism in the PM muscle of TM chickens compared to controls. Expression of pro-angiogenic genes was also triggered in the muscles of these chickens, probably facilitating a vasomotor response and hence heat loss, especially during later heat challenge. In these conditions, TM broilers also greatly modified the overall muscle expression profile compared to controls, probably partly due to epigenetic modifications and active RNA splicing. Metabolic process and stress-responsive pathways were particularly affected in TM birds displaying low body temperatures under heat exposure by improving physiological adaptation processes and preserving cell integrity in the muscle. Further studies are required in order to elucidate the function of new thermotolerance markers in the muscle and in other tissues involved in thermoregulatory and metabolic programming.

## Methods

### Animals, rearing and slaughtering conditions

Chickens were bred at INRA, PEAT (UE1295 Pôle d’Expérimentation Avicole de Tours) in accordance with European Union guidelines for Animal Care. The experiment was approved by the Ethics Committee (“Comité d’Ethique en Expérimentation Animale Val de Loire”, Tours, France, N° 2011-9).

Eggs of Cobb 500 broiler chickens were incubated either in control conditions (C) or in thermally-manipulated conditions (TM) in two semi-commercial automatic incubators (type 360 E, SMA Coudelou, Rochecorbon, France). Control eggs were maintained at 37.8 °C and 56 % relative humidity (RH) during the whole incubation period [48]. Thermal manipulation consisted of incubation at 39.5 °C and 65 % RH for 12 h/24 from Embryonic day E7 to E16 (inclusive). All eggs were turned through 90° every hour. After hatching, male chicks of each treatment were transferred to a single poultry house and reared from d0 to d32. The temperature was gradually decreased from 33 °C at d0 to 21 °C at d25 and remained at 21 °C thereafter. Water and standard feed (21.7 % CP, 2992 kcal/kg from d0 to d28, and 20 % CP, 3100 kcal/kg from d28 to d35) were supplied *ad libitum.* At d32, Control and TM chickens were divided into two groups, i.e. heat-challenged chickens and non-challenged chickens. The challenged group was transferred to a second thermally-controlled room. Following a 2 d adaptation to the new site, heat-challenged chickens were exposed to 32 °C for 5 h on d34 (TMHC and CHC) whereas non-challenged chickens remained under the standard conditions (TM and C). Body temperatures (Tb measured in distal colon) of chickens were measured on d35 at 21 °C and during the heat challenge (32 °C) with an electronic thermometer (Testo 110, Testo, Germany). The effects of the treatments on production traits were reported in a previous study [10]. Briefly, acclimated TM birds were 1.4 % lighter than their control C counterparts at 34 days of age, but they presented less abdominal fat relative to their body weight, and higher breast yield in females. They were also characterized by lower body temperatures and plasma triiodothyronine concentrations from hatching until 28 days of age. The birds included in the present study targeted on gene expression in the muscle tissue were originated from the same experiment. The average body temperatures of the sampled groups were 40.9 ± 0.1 (*n* = 10), 40.7 ± 0.1 (*n* = 11), 42.6 ± 0.2 (*n* = 10) and 42.6 ± 0.2 (*n* = 9), for C, TM, CHC and TMHC groups, respectively [10]. In order to characterize gene expression in the animals better tolerating heat by means of embryo heat acclimation, during the heat challenge 8 male chickens per treatment presenting Tb of 40.6 ± 0.2 °C, 41.1 ± 0.1 °C, 42.5 ± 0.2 °C, 42.8 ± 0.1 °C, for TM, C, TMHC and CHC, respectively, were selected for their low body temperatures for TM and TMHC and high body temperatures for C and CHC. They were slaughtered immediately by cervical dislocation and the breast muscle was excised, snap-frozen and maintained at -80 °C until further analysis.

### RNA isolation and microarray hybridization

Total RNA was extracted from *Pectoralis major* muscle using the RNeasy Midi Kit (Qiagen) according to the manufacturer’s instructions. The concentration and the purity of the RNA samples were measured using a NanoDrop 1000 spectrophotometer (Thermo Scientific), and their integrity (RIN) was evaluated using a Bio-Analyzer 2100 (Agilent Technologies). The RIN values ranged from 8.1 to 9.1.

### Experimental microarray design

**Fig. 6:**
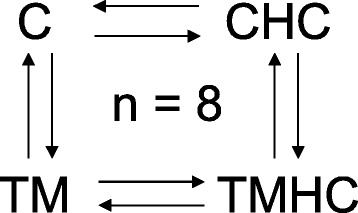
Dye-swap design of the array hybridization of cRNA from *Pectoralis major* muscle of fast-growing chickens. Arrows represent Cy3 vs. Cy5 and reverse arrows represent Cy5 vs. Cy3. Chickens were incubated and reared in standard conditions (Controls, C), thermally manipulated during embryogenesis and reared in standard conditions (TM), incubated in standard conditions and exposed to heat challenge at d 34 (CHC) or thermally manipulated during embryogenesis and exposed to heat challenge at d 34 (TMHC)

A dye swap design was used in the study to analyze four different comparisons (Fig. 6). Briefly, samples from 8 chickens per treatment were compared to samples from two other treatments in a dye swap design using a total of 64 arrays.

### Microarray experiments

Transcriptome profiling was performed using Custom Gene Expression 8 × 60 K *Gallus gallus* array, ID 033525 (Agilent Technologies), that contains the 44 k Agilent probe collection in addition to 5821 customized probes from another collection used in our laboratory and known to be expressed in the chicken liver, adipose tissue and/or *Pectoralis major* muscle, and 623 customized probes corresponding to genes involved in lipid metabolism according to Gene Ontology terms. These two additional probe lists were duplicated on the ID036000 microarray. A total of 64 samples were analysed, corresponding to 8 slides. Cyanine-3 (Cy3) and Cyanine-5 (Cy5)-labelled cRNA was prepared from 100 ng of total RNA using the Two-Color Low Input Quick Amp Labelling Kit (Agilent Technologies). Synthetic RNA (Two-Color RNA Spike-In Kit, Agilent Technologies) was added as an internal quality control. The cRNA obtained was purified using RNeasy Mini Spin columns (Qiagen) and subsequently run on a Bioanalyzer 2100 using RNA 6000 Nano chips (Agilent Technologies) to assess the profiles. Three hundred ng of Cy3-labeled cRNA (specific activity > 9) and 300 ng of Cy5-labeled cRNA (specific activity > 9) were combined for each single array, and the washing steps were then performed according to the manufacturer’s recommendations. The slides were finally scanned using a G2565CA Scanner System (Agilent Technologies), using a scan protocol with a resolution of 3 μm and a 20 bit dynamic range.

The resulting .tiff images were analysed with Feature Extraction Software (Agilent Technologies) using the GE2_107_Sep09 protocol to obtain background-subtracted and spatially-detrended processed signal intensities.

### Filtering and data normalization

The data were filtered on the basis of information on the quality of the spots for the normalization step considering that the signal needed to be more than 1.5 times the background noise. Normalization was performed by global loess function of the Limma package [49].

### Data analysis

Genes differentially expressed (DE) between TM and C, TM and TMHC, C and CHC and TMHC and CHC were identified using a moderated *t*-test where variance was modelized using the shrinkage method in the Limma package under the R statistical environment (http://cran.r-project.org/) from Bioconductor project (http://www.bioconductor.org/).

The raw p-value of each gene was adjusted according to the Benjamini-Hocheberg method to monitor the false discovery rate [50]. Difference in gene expression was judged significant when its adjusted *P*-value was below 0.05.

### Clustering

From the set of genes differentially expressed in at least one of the comparisons, we used the MeV software (MultiExperiment Viewer, http://www.tm4.org/ mev /) to determine the groups of genes sharing the same expression profile. Seven clusters were produced by cutting the dendrogram resulting from the hierarchical clustering using the Pearson correlation coefficient for the metrics between genes and an average linkage for the distance between clusters.

### Real-time RT-PCR assay

RNA samples were reverse-transcribed using RNase H- MMLV reverse transcriptase (Superscript II, Invitrogen, Illkirch, France) and random primers (Promega, Charbonnières les Bains, France). cDNA samples thus obtained were added to SYBR Green I qPCR Master Mix Plus (Eurogentec, Angers, France) as well as specific reverse and forward primers previously designed and validated by sequencing the end-point PCR product to quantify each target gene. Primers for DE genes originating from the microarray analyses are described in Additional file 2. The geNorm analysis was performed on the expression of a set of “standard genes” to find a reliable normalization factor in order to correct expression data of target genes relative to the initial RNA content of samples [51]. In our study, this normalization factor was calculated from the threshold PCR cycles from the genes encoding β-actin, cytochrome B and 18S. Reaction mixtures were incubated in a LightCycler 480 apparatus (Roche Diagnostics, Meylan, France France).

### Functional annotation and promoter analysis

The interpretation of microarray data was performed using Ingenuity Pathway Analysis 7.0 (IPA, Ingenuity Systems Inc., Redwood City, CA) and functional annotation was determined regarding Biological Processes (GO) from the ontology ‘biological process’ from the Gene Ontology Consortium (GO, http://www.geneontology.org/) using Genomatix software. The *P*-values reported were the probabilities (using Fisher’s Exact Test) of finding at least m genes in an input list of length q having annotation A, with q being the number of genes in the input set, m the number of genes from the input set having annotation A assigned, under the assumption that belonging to the input list is independent of having this annotation. The genes included in the analyses were those identified as differentially expressed within one of the four comparisons tested. Using Ingenuity Upstream Regulator Analysis in Ingenuity Pathway Analysis 7.0, for each potential transcriptional regulator (TR), the overlap p‐value called likely upstream regulators based on significant overlap between dataset genes and known targets regulated by a TR.

### Availability of supporting data

The microarray data were deposited in the Gene Expression Omnibus (GEO) public repository http://www.ncbi.nlm.nih.gov/geo. The accession number for the series is GSE70756.

## Additional files

Additional file 1: Upstream regulators found for each cluster. (XLSX 26 kb) Additional file 2: Probes used for real-time PCR validation of a subset ofdifferentially expressed genes identified from the microarray analysis. Abbreviations: ADHFE1: alcohol dehydrogenase, iron containing, 1; ARRDC4: arrestin domain containing 4; B-G, V-region-like B-G antigen-like; BIRC5: baculoviral IAP repeat containing 5; CIB2: calcium and integrin binding family member 2; COL12A1: collagen, type XII, alpha 1; COQ6: coenzyme Q6 homolog, monooxygenase; DUSP8: dual specificity phosphatase 8; EME2: essential meiotic endonuclease 1 homolog 2; ENC1: ectodermal-neural cortex 1; MCAT: malonyl CoA:ACP acyltransferase; MRPL28: 39S ribosomal protein L28; OBSCN: obscurin; POLR2E: polymerase II; PDK4: pyruvate dehydrogenase kinase, isozyme 4; PTEN: phosphatase and tensin homolog; SCN5A: sodium channel, voltage-gated, type V, alpha subunit; SERPINH1: serpin peptidase inhibitor, clade H; SPP1: osteopontin; TBCE: tubulin folding cofactor E; TXNRD2: thioredoxin reductase 2; TTN: titin; TMEM111: transmembrane Protein 111; UBB: ubiquitin B. (DOCX 14 kb)

## Abbreviations

C: control
CHC: control + Heat challenge
DE: differentially expressed
PM: pectoralis major
RT-PCR: real time polymerase chain reaction
TM: thermal manipulation during embryogenesis
TMHC: TM + Heat challenge
