# Supplementary material for: Thermal manipulation of the chicken embryo triggers differential gene expression in response to a later heat challenge
Source: BMC Genomics. 2016 May 4;17:329. doi: 10.1186/s12864-016-2661-y (PMC4855354; doi:10.1186/s12864-016-2661-y)
Supplement: Additional file 2: — Probes used for real-time PCR validation of a subset ofdifferentially expressed genes identified from the microarray analysis. Abbreviations: ADHFE1: alcohol dehydrogenase, iron containing, 1; ARRDC4: arrestin domain containing 4; B-G, V-region-like B-G antigen-like; BIRC5: baculoviral IAP repeat containing 5; CIB2: calcium and integrin binding family member 2; COL12A1: collagen, type XII, alpha 1; COQ6: coenzyme Q6 homolog, monooxygenase; DUSP8: dual specificity phosphatase 8; EME2: essential meiotic endonuclease 1 homolog 2; ENC1: ectodermal-neural cortex 1; MCAT: malonyl CoA:ACP acyltransferase; MRPL28: 39S ribosomal protein L28; OBSCN: obscurin; POLR2E: polymerase II; PDK4: pyruvate dehydrogenase kinase, isozyme 4; PTEN: phosphatase and tensin homolog; SCN5A: sodium channel, voltage-gated, type V, alpha subunit; SERPINH1: serpin peptidase inhibitor, clade H; SPP1: osteopontin; TBCE: tubulin folding cofactor E; TXNRD2: thioredoxin reductase 2; TTN: titin; TMEM111: transmembrane Protein 111; UBB: ubiquitin B. (DOCX 14 kb) [file 12864_2016_2661_MOESM2_ESM.docx]

| **Gene** | **Primers** | **Accession Number** |
| --- | --- | --- |
| **ADHFE1** | Fw: CCCCAGCAGTGTTTGCTTTC | XM_424090.3 |
|  | Rev: TTGATTCTGGCAGTGCGGAT |  |
| **ARRDC4** | Fw: TCCAGCATTACCAGCCAGTT | XM_413881.4 |
|  | Rev: ACAGTCGATTGGTGGTGGAT |  |
| **B-G** | Fw: GAAAAGATGCAGCACTGGCG | XM_424581.3 |
|  | Rev: GCCAGTTCCTCCGTAATCTCA |  |
| **BIRC5** | Fw: CAGTGCTTCTTCTGCCTCAA | NM-204235.2 |
|  | Rev: CATGTTCTCTATCGCGTGCC |  |
| **CIB2** | Fw: TTGCACGTTCTTCACTCGGA | XM_413749.3 |
|  | Rev: GCTCTCTGGGAGCCATTTCA |  |
| **COL12A1** | Fw: GGGACTGGATCTCAAGGACC | NM-205021.1 |
|  | Rev: TCTGGGAATCCTTGGCCATT |  |
| **COQ6** | Fw: CTGGGGCCATGTTTCGATCT | XM_421257.3 |
|  | Rev: TGCTGCACTGAGGTGATGAG |  |
| **DUSP8** | Fw: TCCGACGATGCTTACAGGTT | XM_004941446.1 |
|  | Rev: GACACCTCAGTTGGGTCCTG |  |
| **EME2** | Fw: AGCCTGACCTGAACCAAGTG | XM_414715.3 |
|  | Rev: AGGCTGGGATCTGGGATTC |  |
| **ENC1** | Fw: CCGAAGATGAAAGGCTCGTG | XM_424790.4 |
|  | Rev: GTTCTTCCATCGCCACGTTC |  |
| **MCAT** | Fw: CAGGGCACATACAGGCTTTG | XM_425506.3 |
|  | Rev: ACTGCTGGTTCCATGAGTCG |  |
| **MRPL28** | Fw: ACCATCACCGTCACTATGCG | NM-001031359 |
|  | Rev: GCATCGTTCGCTTCAAGTCC |  |
| **OBSCN** | Fw: TGCTTGCCAGACAAAGAATG | XM_418501.3 |
|  | Rev: CCCTCCTCATGAATCTGGAA |  |
| **POLR2E** | Fw: CATGCAGGAGGAGAACATCA | XM_418224 |
|  | Rev: TGCAGAAACTGCTCCAGGAT |  |
| **PDK4** | Fw: TGACTGGTGCATCCCAAGTAAAG | NM-001199909.1 |
|  | Rev: GGAAGAATTTGCCTGTTTGGAGG |  |
| **PTEN** | Fw: CAAATAAAGACAAAGCCAACAGATA | XM_421555.3 |
|  | Rev: TCGGGATTTGATGGCTCTTC |  |
| **SCN5A** | Fw: AAGCTTGCAAGGAATGCGAAG | XM_001232817.2 |
|  | Rev: AGGCAGGAGTGGCACTAAAT |  |
| **SERPINH1** | Fw: GCCATCTCACTGCCTAAGGT | NM-205291.1 |
|  | Rev: GGTTCCTCATCTCCTCTCGG |  |
| **SPP1** | Fw: GCAGCAGACACAGAATGACC | NM-204535.4 |
|  | Rev: TCTGTGGGGAAGTCTGTGAC |  |
| **TBCE** | Fw: TTTGGCTTGGAGTGGAGTGG | XM_423980 |
|  | Rev: TTGCTTTGTTTGGGCGGATG |  |
| **TXNRD2** | Fw: AGGCTGTGCAGTGTTATGGA | NM-001122691.1 |
|  | Rev: TGAAGGCCAAGGATTCGTTG |  |
| **TTN** | Fw: GGCGAGACTGCAAGATTTTC | XM_421979.3 |
|  | Rev: CGCTTCCTGTCTTCCTTCAG |  |
| **TMEM111** | Fw: CTGCGCTTCAAACCAATGCT | NM-001198612.1 |
|  | Rev: GCTGCCCCTGTCATTTGTTC |  |
| **UBB** | Fw: CGCACTCTGTCCGACTACAA | XM_415105.4 |
|  | Rev: GCCTTCACGTTCTCAATGGT |  |
